# Comparison of Immune Effects Between *Brucella* Recombinant Omp10-Omp28-L7/L12 Proteins Expressed in Eukaryotic and Prokaryotic Systems

**DOI:** 10.3389/fvets.2020.00576

**Published:** 2020-09-18

**Authors:** Lin Zhu, Qiuju Wang, Yujian Wang, Yulin Xu, Duo Peng, He Huang, Liping Hu, Kai Wei, Ruiliang Zhu

**Affiliations:** ^1^Shandong Provincial Key Laboratory of Animal Biotechnology and Disease Control and Prevention, Shandong Agricultural University, Tai'an, China; ^2^Shandong Provincial Engineering Technology Research Center of Animal Disease Control and Prevention, Shandong Agricultural University, Tai'an, China; ^3^Department of Immunology and Infectious Diseases, Harvard T.H. Chan School of Public Health, Boston, MA, United States; ^4^Shandong New Hope Liuhe Co., Ltd., New Hope Group, Qingdao, China; ^5^Animal Disease Prevention and Control Center of Shandong Province, Animal Husbandry and Veterinary Bureau of Shandong Province, Jinan, China

**Keywords:** *Brucella*, subunit vaccine, *Pichia pastoris*, recombinant protein, adjuvant

## Abstract

*Brucella*, a genus of bacteria that causes brucellosis, infects and threatens domestic animals, and humans in endemic areas. Presently, some live attenuated vaccines of *Brucella* are used to immunize livestock; however, these vaccines are pathogenic to humans, can provoke abortion when administered to pregnant livestock, and induce antibodies in vaccinated livestock that affect the diagnosis of field infection. It is, therefore, very important for improving the safety and immune protection effects of *Brucella* vaccine. Currently, recombinant protein-based subunit vaccines are considered promising safe and effective alternatives against brucellosis. Here, we separately expressed the recombinant Omp10-Omp28-L7/L12 proteins of *Brucella* using eukaryotic and prokaryotic expression systems, which were then used as immunogens for evaluating their immune responses. Taishan *Pinus massoniana* pollen polysaccharides (TPPPS), an already verified natural adjuvant, was utilized to evaluate the immune conditioning effect on the recombinant proteins. Antibody levels, spleen lymphocyte proliferation, percentages of CD4^+^ and CD8^+^ T cells, and cytokine secretion in mice were examined after three successive immunizations. The protective effects against *Brucella* challenge were also evaluated in mice, and used a live vaccine as a positive control. The results indicated that the immune responses of the recombinant Omp10-Omp28-L7/L12 protein groups were significantly higher than those of the PBS control group. The recombinant Omp10-Omp28-L7/L12 protein expressed in *Pichia pastoris* (*P. pastoris*) exhibited a slightly higher expression level and immunogenicity than that expressed in *Escherichia coli* (*E. coli*), and the Omp10-Omp28-L7/L12 (*P. pastoris*) + TPPPS group provided the most pronounced immune effect. The protective results showed that the recombinant Omp10-Omp28-L7/L12 proteins expressed in the two expression systems had significantly better protective effects against *Brucella melitensis* challenge compared with the negative control, and the addition of TPPPS adjuvant could significantly improve the protective effects of subunit vaccines. However, we also noticed that all of the evaluated subunit vaccines induced less protection than the *B. melitensis* M5 live vaccine. These results indicate that the combination of recombinant Omp10-Omp28-L7/L12 antigen and TPPPS adjuvant shows potential as an effective brucellosis subunit vaccine, and *P. pastoris* is a preferred expression system to prepare this recombinant subunit antigen.

## Introduction

Brucellosis is a common zoonosis caused by certain species of the *Brucella* genus, which are facultative, intracellular, Gram-negative pathogens (1). *Brucella* leads to abortion in livestock and wildlife and causes multiple illnesses in humans (2). In China, vaccination and eradication are the principles of comprehensive measures to prevent and control brucellosis. Vaccination of susceptible animals is known as essential to control brucellosis in order to protect public health and prevent economic loss (3). In recent decades, China has used several live *Brucella* vaccines. For example, *B. melitensis* M5-90 is one vaccine strain mainly used in goats, whereas *B. melitensis* Rev. 1 is used to protect goats and sheep from *B. melitensis* infection (4). Additionally, strains 19 (S19) (5), 82 (S82) (6), and *B. abortus* RB51 are the three main vaccines believed to protect cattle from *B. abortus* infection. The live brucellosis vaccines can effectively reduce the prevalence of brucellosis in natural hosts, but with known disadvantages: (1) they are unstable and sometimes cause brucellosis associated with vaccines; (2) we cannot distinguish between vaccine reactions and natural infections using antibody detection; (3) some live vaccines can cause allergic reactions similar to strong *Brucella* infection; and (4) some live *Brucella* vaccines cannot be used in pregnant or lactating animals (7). For these reasons, new strategies or improved vaccines against *Brucella* must be developed.

Subunit vaccines that are dependent on antigen identification and expressed in eukaryotic or prokaryotic systems have been widely developed (3, 8). An advantage of recombinant subunit vaccines is that they can not only induce high levels of antibody but also eliminate the safety issues associated with live vaccines (9). Moreover, subunit vaccines can be better controlled because they are more homogenous, therefore, wild and vaccine strain infections can be distinguished by detecting antibodies against subunits and other antigens. Although an adjuvant is usually required with subunit vaccines and they are relatively less protective than conventional vaccines because of their limited antigenic epitopes, subunit vaccines are still believed to be a safer and potential option in the development of the next-generation vaccine (10). Currently, some subunit protein antigens of *Brucella*, particularly outer membrane proteins (Omps) and ribosomal proteins expressed in *E. coli*, serve as vaccine candidates, such as Omp10 (11), Omp16 (12), Omp25 (13), Omp31 (14), Omp2b (15), Omp28 (BP26) (11), and L7/L12 (16, 17). These have all shown good immunoreactivity and protective effects under laboratory conditions (18, 19). Notably, previous studies on *Brucella* subunit vaccines showed that bivalent vaccines (based on recombinant L7/L12-Omp16 or L7/L12-P39 subunits) induce stronger immune responses and better protection against *Brucella* than univalent vaccines (20, 21). Cassataro et al. confirmed a BLS-based chimera decorated with 10 copies of a 27-amino acid epitopes derived from Omp31, which not only induced peptide- and BLS-specific Th1 and cytotoxic T responses but also caused a strong humoral response to the inserted peptide (22). In another study performed by Golshani et al., the rL7/L12-TOmp31 may be a new potential antigen candidate that can be used to develop subunit vaccines against *B. melitensis* and *B. abortus* (16). Nevertheless, other antigen combinations should be tested to determine if they can produce better protection.

In previous studies, the outer membrane proteins Omp10 and Omp28 and ribosomal protein L7/L12 were expressed separately in various systems and were shown good immunogenicity (11, 23). The Omp10 is a surface-exposed lipoprotein, which is an important immunogenic protein of *Brucella* and is strongly conserved and expressed in all known *Brucella* species and their biovars (24). Moreover, Omp10 is also considered to be related to the virulence of *Brucella* (25), and studies have shown that it can induce protective cellular immunity. Omp28 exists in different *Brucella* species, it is a conservative protein. It is a soluble protein that can be released from the inside of the cell to the outside, and it has the advantage of being easy to detect compared with the fixed Omps (26, 27). Not only can it react with IgG antibody, but it can also induce a protective immune response (28). Omp28 has been known as a subunit vaccine candidate and a serological diagnostic antigen (29). The ribosomal protein L7/L12 has been identified as an immunodominant antigen that is considered a priority antigen in the development of *Brucella* subunit vaccines (30), and some studies have been done using L7/L12 in subunit vaccines (23). It has been found that L7/L12 can specifically stimulate monocytes of infected animals and promote the transcription and expression of IFN-γ, which plays an auxiliary role in immune protection (31). L7/L12-based vaccines have the most significant resistance to infection. Thus, in this study, we selected three antigen proteins, Omp10, Omp28, and L7/L12, and studied their compound immunogenicity based on a fusion expression scheme. We expressed the Omp10-Omp28-L7/L12 fusion proteins in the *P. pastoris* eukaryotic expression system and the *E. coli* prokaryotic expression system, to compare the immune effects of the subunit vaccines prepared in these two different expression systems. Subsequently, the immune effects of this triple conjugate immunogen were evaluated in mice.

## Materials and Methods

### Strains and Plasmids

*B. melitensis* 16M strain and *B. melitensis* M5 vaccine were purchased from the China Institute of Veterinary Drug Control (Beijing, China). *P. pastoris* GS115 and plasmid pPIC9 were purchased from Invitrogen (Carlsbad, CA, USA). *E. coli* DH5α and pET-28a (+) vector were preserved in our laboratory. The secondary antibodies were purchased from Abcam (Shanghai, China). All media were prepared according to *Pichia* and *E. coli* expressions manuals. The protection experiment was conducted in a biosafety level 3 (BSL-3) laboratory.

### Construction of the Recombinant Expression Vector

The gene sequences of Omp 10 (GenBank accession number: KF780864.1), Omp 28 (GenBank accession number: JF918758.1), L7/L12 (GenBank accession number: EF173477.1) of *Brucella* strains were retrieved from NCBI, and *BamH I, Xhol I* (*E. coli*) and *Xhol I, Not I* (*P. pastoris*) were used, respectively, in the two expression systems. The designed Omp10-Omp28-L7/L12 fusion gene sequence (1,623 bp) was synthesized after codon optimization from GENEWIZ, with the Omp10, Omp28, and L7/L12 genes being 375, 751, and 387 bp, respectively. Thus, primers for Omp10, Omp28, and L7/L12 were designed (Tables S1, S2). In addition, we predicted the three-dimensional structure of the fusion protein using the homology modeling method SWISS-MODEL (32–34). The expected size of the recombinant protein Omp10-Omp28-L7/L12 was 55.4 kDa. The fused gene was cloned into the pET-28a (+) vector and the pPIC9 vector separately [pET-28a (+)-Omp10-Omp28-L7/L12 and pPIC9-Omp10-Omp28-L7/L12] and confirmed by sequencing (TSINGKE, Beijing). The recombinant plasmids were transformed into *E. coli* DH5α strain to expand the yield.

### Construction, Expression, Purification, and Identification of the Recombinant Proteins

The fused Omp10-Omp28-L7/L12 fragment was first artificially synthesized after codon optimization. The PCR product was cloned into the prokaryotic expression vector pET-28a (+). The constructed recombinant plasmids were verified by sequencing and then transformed into competent *E. coli* BL21 (DE3) to obtain the pET-28a (+)-Omp10-Omp28-L7/L12, pET-28a (+)-Omp10, pET-28a (+)-Omp28, and pET-28a (+)-L7/L12 *E. coli* transformants. Induction was performed using 1.0 mmol/L IPTG at different times and the inclusion bodies were extracted via sonication. The transformants were cultured with IPTG while shaking at 37°C for 6 h. The bacterial suspensions were centrifuged at 4°C and the bacterial lysates were collected at 1, 3, 5, and 6 h after IPTG induction.

Meanwhile, the PCR product was cloned into the eukaryotic expression vector pPIC9. The constructed recombinant plasmid pPIC9-Omp10-Omp28-L7/L12 was verified by sequencing and then transformed into competent *P. pastoris* GS115 to obtain the transformants. The blank plasmid pPIC9 was transformed into *P. pastoris* as a negative control. Methanol was used to induce protein expression. After inducing methanol for 24, 48, 72, and 96 h, centrifuged and collected the culture supernatant.

Recombinant proteins were purified using the ProteinIso™ Ni-NTA Resin kit (TRANS, Beijing, China) and identified by SDS-PAGE and western blot analysis in accordance with a previous study. Five percentage of concentration gel and 12% of separation gel were chosen to SDS-PAGE identification. Determined protein concentration using the Easy II Protein Quantitative Kit (BCA) (TRANS, Beijing, China).

For SDS-PAGE and western blot analysis, mouse polyclonal antibodies were prepared by multiple immunizations with the Omp10, Omp28, and L7/12 proteins separately expressed in the *E. coli* prokaryotic expression system. We used LE Buffer (100 mM Na_2_HPO_4_, 10 mM Tris-CI, 8 M urea) to dissolve the inclusion bodies. Then the expressed recombinant Omp10-Omp28-L7/L12 proteins within the two expression systems were identified by western blot using mouse anti-His tag antibody, mouse anti-Omp10 polyclonal antibody, mouse anti-Omp28 polyclonal antibody, and mouse anti-L7/L12 polyclonal antibody.

### Preparation of the Vaccines

We added 10% glycerin, 3% sucrose, and 3% sorbitol to the expressed recombinant Omp10-Omp28-L7/L12 proteins as protective agents. TPPPS has been proven to be an effective adjuvant for improving the immune effects of vaccines (35–38) and was preserved in our laboratory. TPPPS has been confirmed to elicit better humoral and cellular immunity and enhance the stimulation ability of the vaccines (39, 40). Moreover, TPPPS has been shown to be an effective adjuvant for promoting immune responses and improving the immune system (35, 37, 41, 42). The purified recombinant Omp10-Omp28-L7/L12 proteins were mixed with a dose of 50 mg/mL TPPPS at a ratio of 1:1 to a final concentration of 1 mg/mL. Stability and sterility tests were then conducted to evaluate the recombinant subunit vaccines.

### Animal Experiments

The total of 270 5–6 weeks old pathogen-free (SPF) BALB/c female mice, were randomly divided into nine groups (groups I-IX) of 30 individuals and reared under identical environmental conditions. Ambient conditions were set at 22–25°C and 32–42% relative humidity and the air entering the isolators was filtered. Group I mice were vaccinated with 0.1 mg of pure Omp10-Omp28-L7/L12 (*P. pastoris*) subunit vaccine (Group I), group II mice were vaccinated with 0.1 mg of TPPPS adjuvant Omp10-Omp28-L7/L12 (*P. pastoris*) subunit vaccine (Group II), group III mice were vaccinated with 0.1 mg of pure Omp10-Omp28-L7/L12 (*E. coli*) subunit vaccine (Group III), group IV mice were vaccinated with 0.1 mg of phosphate buffered saline (PBS) (Group IV), group V mice were vaccinated with 0.1 mg of pure Omp10 subunit vaccine (Group V), group VI mice were vaccinated with 0.1 mg of pure Omp28 subunit vaccine (Group VI), group VII mice were vaccinated with 0.1 mg of pure L7/L12 subunit vaccine (Group VII), group VIII mice were vaccinated with 0.1 mg of TPPPS adjuvant Omp10-Omp28-L7/L12 (*E. coli*) subunit vaccine (Group VIII), group IX mice were vaccinated with 0.1 mg of *B. melitensis* M5 live vaccine (Group IX) and then revaccinated at 7 and 14 days post-vaccination (dpv). At 0, 14, 28, 42, and 56 dpv, five mice per group were randomly selected to assess the relevant immune indexes. Mice were starved for 12 h before sampling. All mice were vaccinated by hypodermic route.

### Detection of Serum Antibody Levels and Cytokine Concentrations

To determine serum reactivity against Omp10, Omp28, L7-L12, and Omp10-Omp28-L7/L12, immune serum IgG, IgG1, and IgG2a levels were detected using indirect enzyme-linked immunosorbent assay (ELISA). The high binding 96 Well-Single-Break Strip Plates were coated with purified proteins (10 μg/ml) at 100 μl/well-overnight at 4°C. Horseradish peroxidase conjugated goat anti-mouse IgG, IgG1, or IgG2a antibodies were used to measure. Five mice serum samples were randomly sampled from each group at different times from mice orbit. The absorbance at 450 nm (OD_450_) of each well was detected at 30 min after substrate addition. The secretion of IL-2, IL-4, and IFN-γ cytokines were tested using mouse IL-2, IL-4, and IFN-γ ELISA kits (Langdon Bio-technology Co. Ltd., Shanghai), with the optical density at 450 nm were measured using a microplate reader.

### Proliferation of Peripheral Blood Lymphocytes

Randomly collected fresh anticoagulated peripheral blood samples from five mice (1.0 mL/mouse) in each group at different times from mice orbit and mixed with 1.0 mL RPMI-1640. Then added 2.0 mL of the mixture to 5.0 mL of lymphocyte isolation medium (Solarbio, China) to isolate the lymphocytes (43). Centrifuged at 4°C for 20 min at 1,500 rpm to separate the lymphocytes in the middle layer of milky white floc and collected them. Then washed them with lymphocyte washing solution twice. Proliferation of the lymphocytes was detected by the MTT method described in previous studies (44). Adjusted the density of lymphocytes to 1 × 10^6^/mL. In order to avoid edge effects, 1,640 cell culture solutions were added to the edge of the 96-well-plate before adding to the 96-well-plate. Subsequently, 100 μL of the adjusted lymphocyte suspensions were added to every well of a 96-well cell culture plate, and each sample was repeated 6 wells. In addition, added ConA solutions to the first 3 wells to a final concentration of 5 μg/mL, and then added an equal volume of 1,640 medium to the last 3 wells as a control. After sealing and labeling the 96-well-plate, we placed it in an incubator and kept it for 12 h. Observed cell growth status. After the bottom of the cell culture plate was covered with cells, added 10 μL MTT to each well to make the final MTT concentration reach 5 mg/mL, incubated for 4 h, discarded the culture medium, and added 100 μL DMSO to each well. After mixing, the OD value at 490 nm was detected using a microplate reader.

Lymphocyte transformaton rate (LTR) = (mean of Con A stimulation group-mean of non-Con A stimulation group)/mean of non-Con A stimulation group.

### Measurement of CD4^+^ and CD8^+^ T Lymphocytes Counts in Peripheral Blood

Randomly collected fresh anticoagulated peripheral blood samples from five mice (1.0 mL/mouse) in each group at different times from mice orbit and mixed with 1.0 mL PBS. Then added 2.0 mL of the mixture to 5.0 mL of lymphocyte isolation medium (Solarbio, China). The mixture was centrifuged and lymphocytes were separated and isolated. Centrifuged at 4°C for 20 min at 1,500 rpm to separate the lymphocytes in the middle layer of milky white floc and collect them. Then washed them with lymphocyte washing solution twice. Then added the FITC anti-mouse CD4^+^ and APC anti-mouse CD8^+^ dyes to samples to be tested, and placed them in a 4°C refrigerator for 20 min after taking measures to prevent light. Measured the percentages of CD4^+^ and CD8^+^ T lymphocytes using flow cytometry (Guava Easy Cyte Mini, USA) (45).

### Protection Experiments

To evaluate the protective effects of the subunit vaccines against *B. melitensis* challenge, five mice per group were challenged intraperitoneally (*i.p*.) with 5 × 10^5^ CFU of *B. melitensis* 16M strain in 100 μL of PBS, 4 weeks after the last immunization. Notably, a positive control group was added with another five mice that were vaccinated intraperitoneally with 5 × 10^4^ CFU of *B. melitensis* M5 live vaccine 4 weeks before the challenge. Mice were euthanized at 4 weeks post-challenge and their spleens were removed aseptically. Determine the number of *B. melitensis* (CFU) in each spleen, and express the results as the mean log_10_ CFU ± standard deviation (SD) of each group. The log unit of protection was obtained by subtracting the mean log_10_ CFU of the vaccinated group from the mean log_10_ CFU of the PBS control group.

### Statistical Analyses

Data were expressed as mean ± standard deviation. Duncan's multiple-range test was performed to analyze differences among groups using SPSS 17.0. Values of *P* < 0.05 were considered statistically significant.

## Results

### Construction, Expression, and Identification of Recombinant Proteins

**Figure 1:**
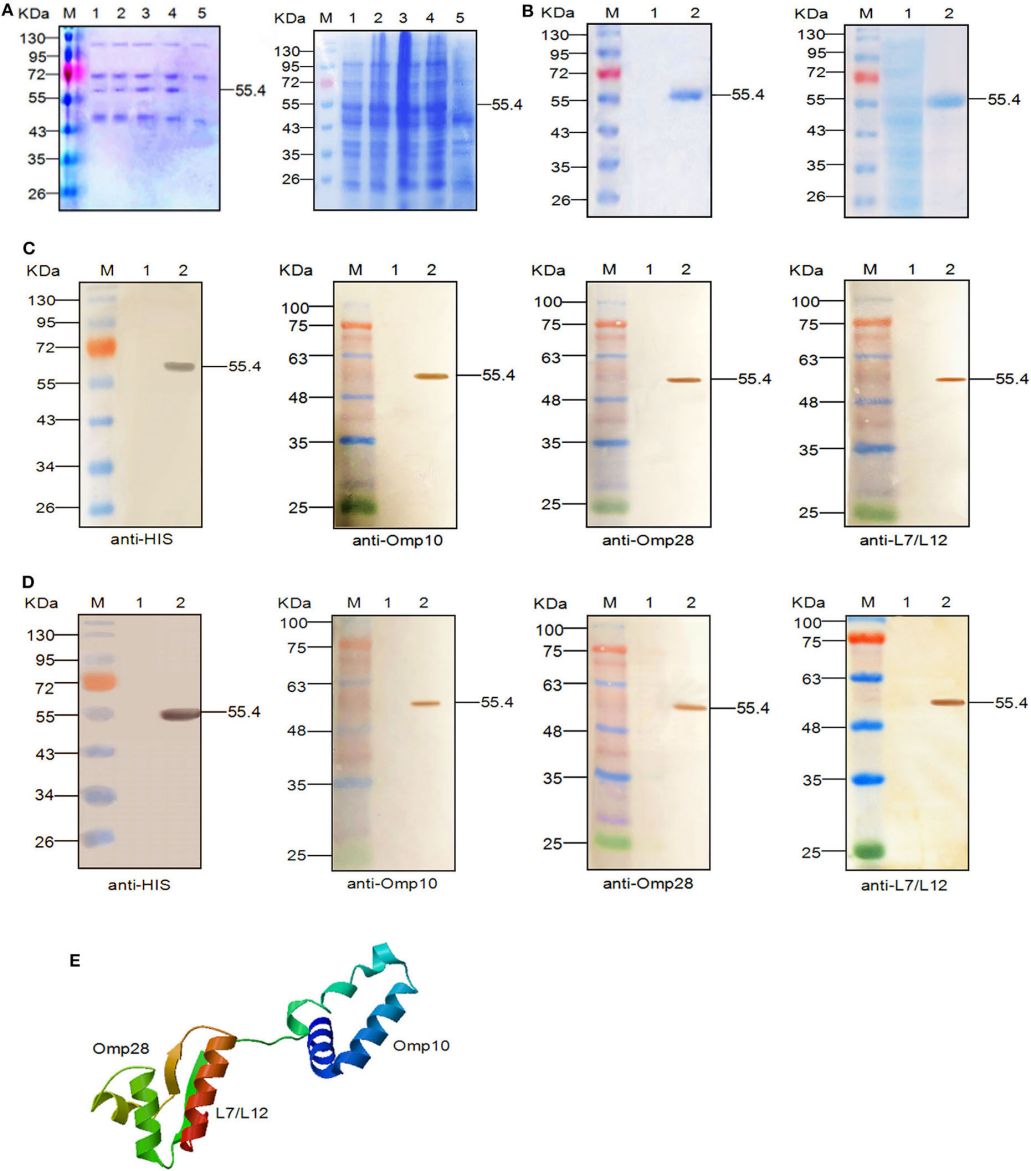
Expression, SDS-PAGE identification, and western blot analysis of the recombinant proteins. **(A)** SDS-PAGE identification of the recombinant Omp10-Omp28-L7/L12 protein (55.4 kDa) at different induction times. Left: M, Page ruler pre-stained protein ladder; lanes 1–4, culture supernatant of *P. pastoris* transformed with the recombinant plasmids after 24, 48, 72, and 96 h of methanol induction; lane 5, culture supernatant of *P. pastoris* transformed with blank pPIC9 vector (negative control). Right: M, Page ruler pre-stained protein ladder; lanes 1–4, expression products of *E. coli* BL21 (DE3) transformed with recombinant plasmids after 1, 3, 5, and 6 h of IPTG induction; lane 5, expression product of *E. coli* BL21 (DE3) transformed with blank pET28a (+) vector (negative control). **(B)** Purification of the fused Omp10-Omp28-L7/L12 protein (55.4 kDa). Left: M, Page ruler pre-stained protein ladder; lane 1, culture supernatant after column chromatography; lane 2, purified Omp10-Omp28-L7/L12 protein (*P. pastoris*). Right: M, Page ruler pre-stained protein ladder; lane 1, unpurified protein product; lane 2, purified Omp10-Omp28-L7/L12 protein (*E. coli*). **(C)** Western blot identification of the recombinant proteins (55.4 kDa) using anti-His tag antibody, mouse anti-Omp10 polyclonal antibody, mouse anti-Omp28 polyclonal antibody, and mouse anti-L7/L12 polyclonal antibody. M, protein molecular size page ruler; lane 1, culture supernatant of *P. pastoris* transformed with blank pPIC9 vector (negative control); lane 2, culture supernatant of *P. pastoris* transformed with the recombinant plasmids at 96 h post-induction. **(D)** Western blot identification of the recombinant proteins (55.4 kDa) using anti-His tag antibody, mouse anti-Omp10 polyclonal antibody, mouse anti-Omp28 polyclonal antibody, and mouse anti-L7/L12 polyclonal antibody. M, protein molecular size page ruler; lane 1, expression products of *E. coli* BL21 (DE3) transformed with blank pET28a (+) vector (negative control); lane 2, expression products of *E. coli* BL21 (DE3) transformed with the recombinant plasmids at 6 h post-induction. **(E)** Predicted 3D structure of the recombinant protein.

Cloned the Omp10-Omp28-L7/L12 fusion gene correctly into the eukaryotic expression vector pPIC9 after sequencing identification. After induced with methanol, using SDS-PAGE, we observed 55.4 kDa protein bands in the culture supernatant of recombinant pPIC9-Omp10-Omp28-L7/L12 transformants at different times (Figure 1A, left). After 72 h of culture, protein was measured in the supernatant and the highest protein concentration (9.4 mg/L) was observed at 96 h. Meanwhile, cloned the Omp10-Omp28-L7/L12 fusion gene correctly into the prokaryotic expression vector pET-28a (+) after sequencing identification. SDS-PAGE showed that the recombinant pET-28a (+)-Omp10-Omp28-L7/L12 transformants corresponded to the expressed protein bands of 55.4 kDa (Figure 1A, right). The peak protein concentration (7.2 mg/L) occurred at 6 h.

Single-protein bands with a molecular weight of 55.4 kDa were also detected in the SDS-PAGE results after purification (Figure 1B). After western blot analysis, we observed that the single reaction bands (55.4 kDa) corresponded to the bands in the SDS-PAGE, indicating the expression of the recombinant Omp10-Omp28-L7/L12 proteins and their specific reactivity to several specific antibodies mentioned above (Figures 1C,D). In addition, we obtained the three-dimensional structural model of the fusion protein Omp10-Omp28-L7/L12 through the homology modeling function of the SWISS-MODEL website. The results showed that the folding of each protein segment maintained their individual structures by the connection of a flexible peptide (Figure 1E).

### Comparison of Immune Effects by Measuring Serum Antibody Levels

**Figure 2:**
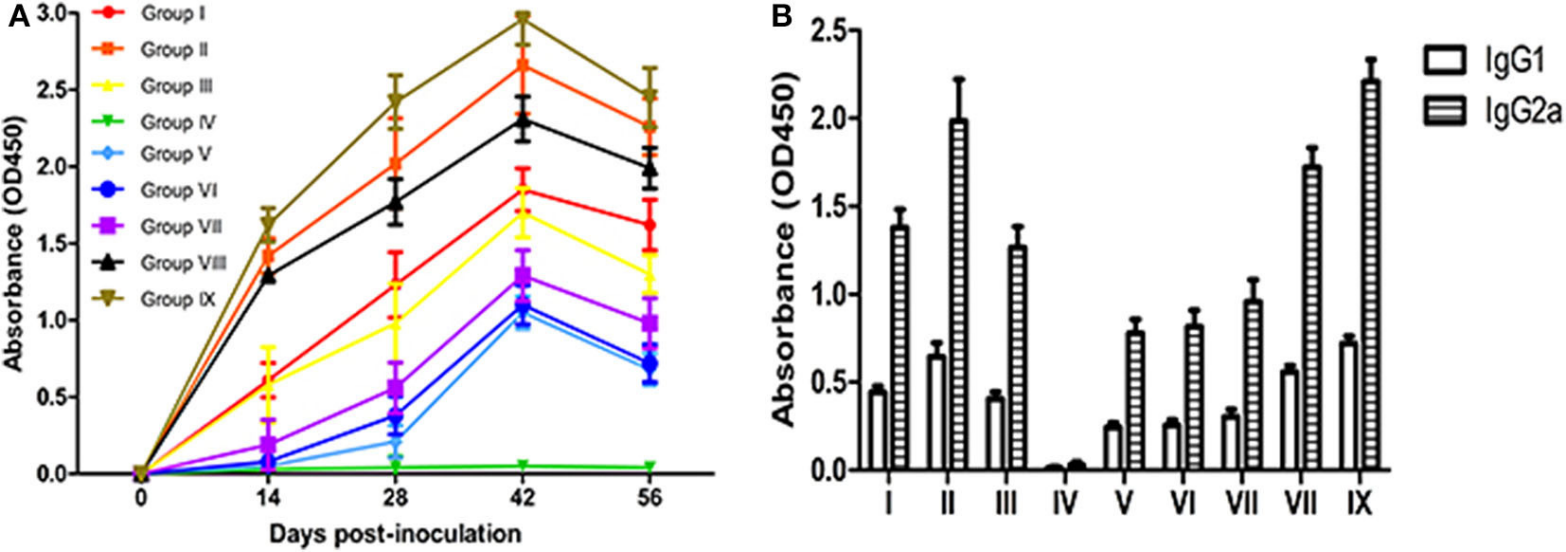
Changes in serum antibody levels in mice. Mice from the nine groups were vaccinated with pure Omp10-Omp28-L7/L12 (*P. pastoris*) subunit vaccine (Group I), TPPPS adjuvant Omp10-Omp28-L7/L12 (*P. pastoris*) subunit vaccine (Group II), pure Omp10-Omp28-L7/L12 (*E. coli*) subunit vaccine (Group III), PBS (Group IV), pure Omp10 subunit vaccine (Group V), pure Omp28 subunit vaccine (Group VI), pure L7/L12 subunit vaccine (Group VII), TPPPS adjuvant Omp10-Omp28-L7/L12 (*E. coli*) subunit vaccine (Group VIII), or B. melitensis M5 live vaccine (Group IX) at 5–6 weeks old. Serums were collected at 0, 14, 28, 42, and 56 dpv. **(A)** The IgG was then determined using indirect ELISA. **(B)** Specific antibody IgG1 and IgG2a levels were measured by ELISA in sera obtained at 45 days after the first immunization. All values shown are means ± SD from five independent experiments.

In order to compare the immune effects of vaccines, it is essential to measure the level of antibodies induced by vaccines. The dynamic changes in serum antibody levels in each group are shown in Figure 2A. A higher OD value represents a higher antibody level detected with the indirect enzyme-linked immunosorbent assay. The results show that the antibody levels of vaccinated mice were significantly higher than levels in the PBS control group mice at 14–56 dpv (*P* < 0.05). Although the antibody levels of the live vaccine group (Group IX) were higher than all experimental groups. The difference between groups Omp10-Omp28-L7/L12 (*P. pastoris*) (Group I) and Omp10-Omp28-L7/L12 (*E. coli*) (Group III) was not significant (*P* > 0.05). And the antibody levels in single proteins groups were lower than fusion protein groups. In addition, the antibody levels in group Omp10-Omp28-L7/L12 + TPPPS (Group II and Group VIII) were significantly higher compared to the other vaccinated protein groups at 14–56 dpv (*P* < 0.05). In order to analyze the potential role of IgG subtypes in the prevention of *Brucella* infection, we examined the proportion of Th1-related IgG2a and Th2-related IgG1 in the total serum IgG of each group by ELISAs at 45 days after the first immunization. The specific IgG2a titers were higher than the specific IgG1 titers in all vaccinated groups indicating a shift toward a Th1 type of response (Figure 2B). Omp10 (Group V), Omp 28 (Group VI) and L7 / L12 (Group VII) groups also had higher IgG2a titers than IgG1 titers, indicating that a single protein can also shift the immune response to Th1 but with lower titers. The results showed that the recombinant *Brucella* protein Omp10-Omp28-L7/L12 had good immunogenicity, and TPPPS could effectively promote antibody production in mice given recombinant subunit vaccine.

### Comparison of Immune Effects by Measuring Cytokine Secretion

**Figure 3:**
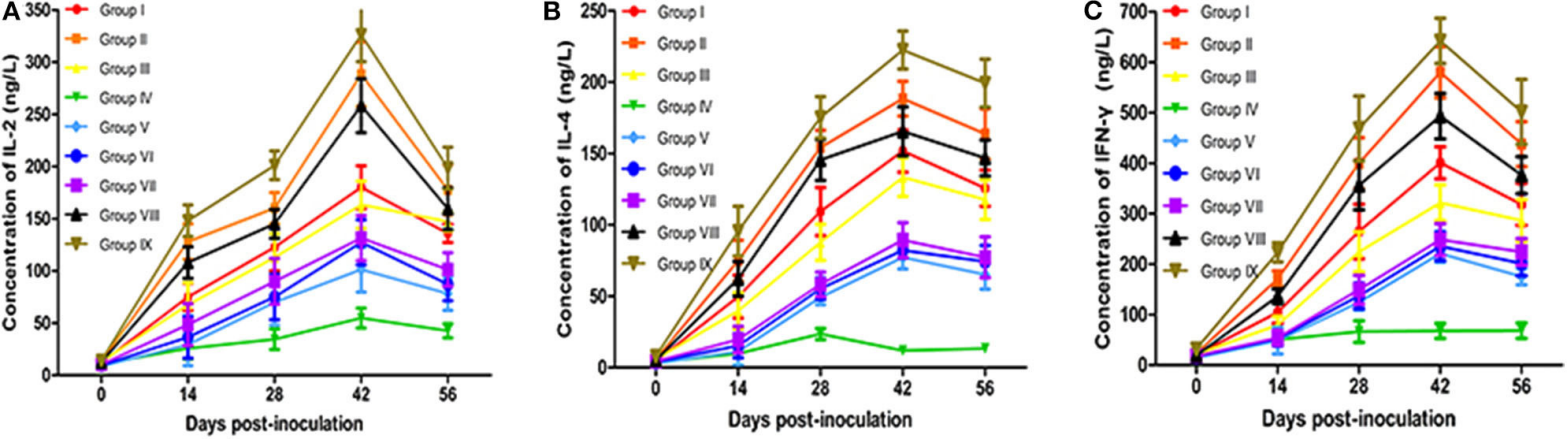
Changes in cytokines in mice. Mice from the nine groups were vaccinated with pure Omp10-Omp28-L7/L12 (*P. pastoris*) subunit vaccine (Group I), TPPPS adjuvant Omp10-Omp28-L7/L12 (*P. pastoris*) subunit vaccine (Group II), pure Omp10-Omp28-L7/L12 (*E. coli*) subunit vaccine (Group III), PBS (Group IV), pure Omp10 subunit vaccine (Group V), pure Omp28 subunit vaccine (Group VI), pure L7/L12 subunit vaccine (Group VII), TPPPS adjuvant Omp10-Omp28-L7/L12 (*E. coli*) subunit vaccine (Group VIII), or B. melitensis M5 live vaccine (Group IX) at 5–6 weeks old. Serums were collected at 0, 14, 28, 42, and 56 dpv. Secretion of IL-2 **(A)**, IL-4 **(B)**, and IFN-γ **(C)** was detected using mouse IL-2, IL-4, and IFN-γ ELISA kits. All values shown are means ± SD from five independent experiments.

Cytokines are important in development, maturation, differentiation, and activation of immune cells. They help to regulate the type and intensity of immune responses and are essential for the resistance of infections (46). Cytokines IL-2 and IFN-γ play an important role in cell-mediated immune response, while IL-4 promotes antibody production and acts on humoral immune response. The serum levels of IL-2, IL-4, and IFN-γ in all groups are displayed in Figures 3A–C. The secretion of IL-2, IL-4, and IFN-γ in vaccinated mice increased significantly compared with the PBS control group, reaching peak values at 42 dpv (*P* < 0.05). Although the contents of IL-2, IL-4, and IFN-γ of live vaccine group (Group IX) were higher than all experimental groups. The contents of IL-2, IL-4, and IFN-γ in the Omp10-Omp28-L7/L12 + TPPPS group (Group II and Group VIII) were significantly higher than those in Omp10-Omp28-L7/L12 (*P. pastoris*) group (Group I) and Omp10-Omp28-L7/L12 (*E. coli*) group (Group III) (*P* < 0.05). The TPPPS adjuvant group showed higher contents of IL-2, IL-4, and IFN-γ than the other groups at 14–56 dpv (*P* < 0.05). And the contents of IL-2, IL-4, and IFN-γ in single proteins groups were lower than fusion protein groups. These results indicate that TPPPS could significantly promote the productions of IL-2, IL-4, and IFN-γ in vaccinated mice.

### Comparison of Immune Effects by Measuring Lymphocyte Proliferation

**Figure 4:**
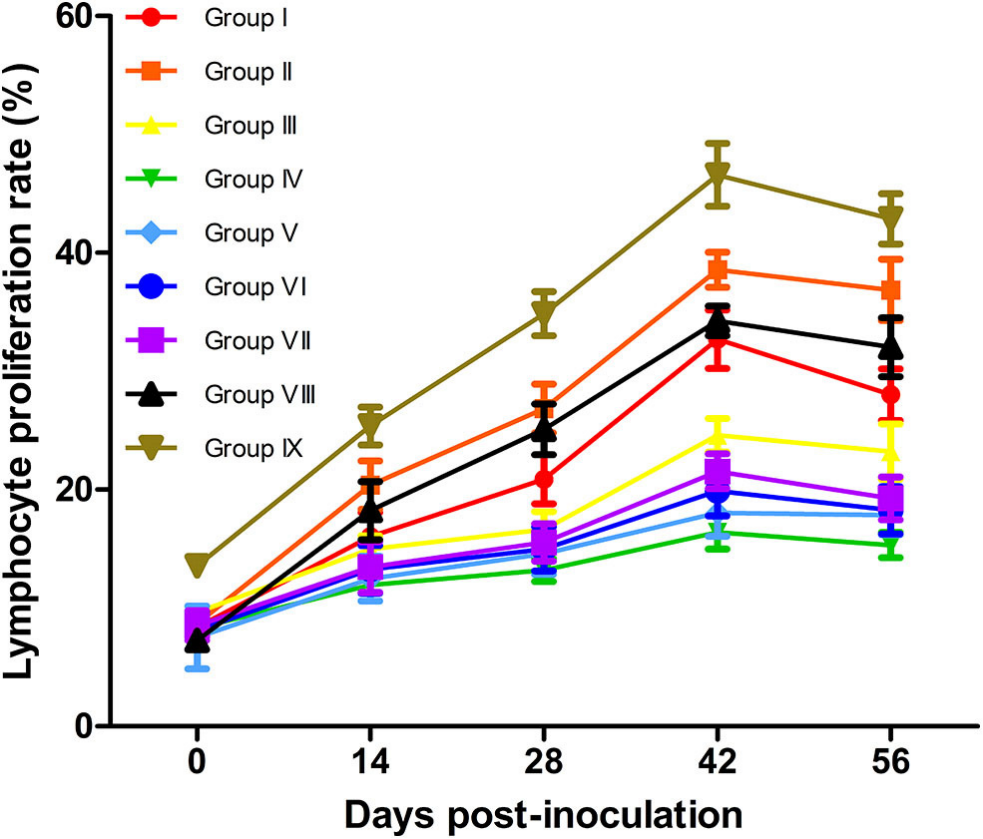
Changes in T lymphocyte proliferation ratio in mice. Mice from the nine groups were vaccinated with pure Omp10-Omp28-L7/L12 (*P. pastoris*) subunit vaccine (Group I), TPPPS adjuvant Omp10-Omp28-L7/L12 (*P. pastoris*) subunit vaccine (Group II), pure Omp10-Omp28-L7/L12 (*E. coli*) subunit vaccine (Group III), PBS (Group IV), pure Omp10 subunit vaccine (Group V), pure Omp28 subunit vaccine (Group VI), pure L7/L12 subunit vaccine (Group VII), TPPPS adjuvant Omp10-Omp28-L7/L12 (*E. coli*) subunit vaccine (Group VIII), or *B. melitensis* M5 live vaccine (Group IX) at 5–6 weeks old. Peripheral blood was collected from each animal at 0, 14, 28, 42, and 56 dpv. The ratio of T lymphocyte proliferation in the same column was compared using Duncan's multiple-range tests. All values shown are means ± SD from five independent experiments.

Proliferation ratios are commonly used to assess cellular immunity (47). The results of lymphocyte proliferation rates are shown in Figure 4. At 14–56 dpv, lymphocyte transformation rates (LTRs) in vaccinated mice were significantly higher than those in the PBS-treated (Group IV) mice (*P* < 0.05). Although the LTRs of live vaccine group (Group IX) were higher than all experimental groups. Notably, the LTRs in the Omp10-Omp28-L7/L12 (*P. pastoris*) (Group I) mice were higher than those in the Omp10-Omp28-L7/L12 (*E. coli*) (Group III) mice, but the difference was not significant (*P* > 0.05). Furthermore, the Omp10-Omp28-L7/L12 + TPPPS (Group II and Group VIII) mice showed significantly higher LTRs than the Omp10-Omp28-L7/L12 (*P. pastoris*) (Group I) and the Omp10-Omp28-L7/L12 (*E. coli*) (Group III) mice at 14–56 dpv (*P* < 0.05). And the LTRs in single protein groups were lower than fusion protein groups. These results suggest that TPPPS significantly promotes cellular and humoral immune responses, induced by the Omp10-Omp28-L7/L12 antigen.

### Comparison of Immune Effects by Measuring Lymphocyte Subset Ratios

The levels of CD4^+^ and CD8^+^ T lymphocytes directly reflect the immune function of animals and are the clearest indicators of immune system damage in infected animals (48). The changes in CD4^+^ and CD8^+^ T lymphocyte counts in peripheral blood are shown in Tables 1, 2, respectively. Although the levels of CD4^+^ and CD8^+^ T lymphocytes of live vaccine group (Group IX) were higher than all experimental groups. The levels of CD4^+^ and CD8^+^ T lymphocytes in vaccinated mice were significantly higher than those in the PBS-treated (Group IV) mice at 14–56 dpv (*P* < 0.05). The levels of CD4^+^ and CD8^+^ T lymphocytes in Omp10-Omp28-L7/L12 (*P. pastoris*) (Group I) mice were higher than those in Omp10-Omp28-L7/L12 (*E. coli*) (Group III) mice, but the difference was not significant (*P* > 0.05). Furthermore, the percentages of CD4^+^ and CD8^+^ T lymphocytes in Omp10-Omp28-L7/L12 + TPPPS (Group II and Group VIII) mice were significantly higher than those in the Omp10-Omp28-L7/L12 (*P. pastoris*) (Group I) and the Omp10-Omp28-L7/L12 (*E. coli*) (Group III) mice at 14–56 dpv (*P* < 0.05). And the levels of CD4^+^ and CD8^+^ T lymphocytes in single proteins groups were lower than fusion protein groups. These results suggest that TPPPS significantly increases the levels of CD4^+^ and CD8^+^ T lymphocytes in the peripheral blood of vaccinated mice.

**Table 1:** Changes in CD4^+^ T lymphocyte counts in the peripheral blood.

**Group^A^**	**Days post-vaccination^B^**				
	**0**	**14**	**28**	**42**	**56**
I	9.69 ± 0.53^a^	15.74 ± 1.28^b^	22.87 ± 2.86^b, c^	30.28 ± 4.07^c^	23.88 ± 1.95^c^
II	8.63 ± 1.25^a^	20.54 ± 2.85^c^	29.34 ± 3.44^d^	38.03 ± 1.61^d^	32.49 ± 2.69^d^
III	9.81 ± 0.80^a^	15.33 ± 0.69^b^	19.54 ± 1.73^b^	27.06 ± 2.11^c^	18.97 ± 1.75^c^
IV	8.61 ± 1.33^a^	9.88 ± 1.01^a^	10.70 ± 1.16^a^	11.02 ± 1.29^a^	11.43 ± 0.85^a^
V	9.26 ± 0.88^a^	10.72 ± 0.68^a^	12.41 ± 1.89^a^	18.82 ± 2.96^b^	13.01 ± 1.74^b^
VI	9.24 ± 0.33^a^	11.70 ± 1.25^a^	13.39 ± 1.65^a^	19.80 ± 2.21^b^	13.99 ± 1.85^b^
VII	9.56 ± 1.33^a^	13.02 ± 0.98^ab^	14.71 ± 1.27^a^	21.12 ± 1.75^b^	15.31 ± 1.08^b^
VIII	9.74 ± 0.95^a^	20.13 ± 3.09^c^	26.02 ± 2.21^cd^	34.81 ± 1.48^d^	27.58 ± 2.50^d^
IX	9.85 ± 0.54^a^	21.40 ± 3.66^c^	36.00 ± 1.82^e^	47.54 ± 0.72^e^	38.40 ± 1.99^e^

A *Group names represent mice in these groups vaccinated with pure Omp10-Omp28-L7/L12 (P. pastoris) vaccine (Group I), TPPPS adjuvant Omp10-Omp28-L7/L12 (P. pastoris) vaccine (Group II), pure Omp10-Omp28-L7/L12 (E. coli) (Group III), PBS (Group IV), Omp10 (Group V), Omp28 (Group VI), L7/L12 (Group VII), TPPPS adjuvant Omp10-Omp28-L7/L12 (E. coli) (Group VIII) or B. melitensis M5 (Group IX) at 5–6 weeks old*.

B *CD4^+^ T lymphocyte counts in the same column were compared using Duncan's multiple-range tests. Different superscript lowercase letters indicate significant differences (P < 0.05). Data are expressed as mean percentage ± SD*.

**Table 2:** Changes in CD8^+^ T lymphocyte counts in the peripheral blood.

**Group^A^**	**Days post-vaccination^B^**				
	**0**	**14**	**28**	**42**	**56**
I	6.55 ± 0.36^a, b, c^	10.64 ± 0.87^c, d^	12.84 ± 1.05^c^	17.20 ± 2.31^d^	14.47 ± 1.81^d, e^
II	5.53 ± 0.81^a^	13.78 ± 2.47^e^	17.47 ± 1.45^d^	21.61 ± 0.91^e^	18.57 ± 2.18^f^
III	5.87 ± 0.48^a, b^	9.00 ± 0.46^b, c^	10.20 ± 0.94^b^	15.38 ± 1.20^d^	12.37 ± 1.10^c, d^
IV	5.94 ± 0.92^a, b^	6.15 ± 0.90^a^	6.14 ± 0.45^a^	6.26 ± 0.73^a^	6.77 ± 0.73^a^
V	6.52 ± 0.36^a, b, c^	7.32 ± 0.55^a, b^	8.52 ± 1.07^b^	9.69 ± 1.14^b^	8.69 ± 1.13^a, b^
VI	6.42 ± 0.22^a, b, c^	7.88 ± 0.87^a, b^	8.74 ± 0.62^b^	11.26 ± 0.89^b, c^	9.25 ± 1.05^a, b^
VII	6.80 ± 0.21^a, b, c^	8.44 ± 0.39^a, b, c^	9.63 ± 0.51^b^	13.21 ± 1.03^c^	10.81 ± 1.34^b, c^
VIII	7.19 ± 0.55^c^	12.15 ± 1.76^d, e^	14.83 ± 1.34^c^	19.78 ± 0.84^e^	16.47 ± 1.41^e, f^
IX	8.54 ± 1.13^d^	18.42 ± 2.35^f^	21.44 ± 2.12^e^	28.44 ± 1.08^f^	22.01 ± 1.81^g^

A *Group names represent mice in these groups vaccinated with pure Omp10-Omp28-L7/L12 (P. pastoris) vaccine (Group I), TPPPS adjuvant Omp10-Omp28-L7/L12 (P. pastoris) vaccine (Group II), pure Omp10-Omp28-L7/L12 (E. coli) (Group III), PBS (Group IV), Omp10 (Group V), Omp28 (Group VI), L7/L12 (Group VII), TPPPS adjuvant Omp10-Omp28-L7/L12 (E. coli) (Group VIII), or B. melitensis M5 (Group IX) at 5–6 weeks old*.

B *CD8^+^ T lymphocyte counts in the same column were compared using Duncan's multiple-range tests. Different superscript lowercase letters indicate significant differences (P < 0.05). Data are expressed as mean percentage ± SD*.

### Protective Effects of *Brucella* Subunit Vaccines

In this experiment, the number of bacteria in the spleen of the immunized mice was significantly reduced compared to the mice inoculated with PBS, and the vaccine efficacy was expressed as the log_10_ of protection. As shown in Table 3, the immunity-induced protection levels of all tested vaccines were significantly higher compared to the PBS control group (*P* < 0.05). The recombinant Omp10-Omp28-L7/L12 (*P. pastoris*) protein induced a level of protection 1.49 logs against the challenge, whereas the recombinant Omp10-Omp28-L7/L12 (*E. coli*) protein induced a level of protection 0.98 logs against the challenge, with no significant difference between the two groups. Among the test vaccines, the level of protection (2.41 logs) in Omp10-Omp28-L7/L12 + TPPPS (Group II and Group VIII) was significantly higher than that in the other protein groups (*P* < 0.05). And the levels of protection in single protein groups were lower than fusion protein groups. However, we also noticed that all of the evaluated subunit vaccines induced lower protective levels than the *B. melitensis* M5 live vaccine (2.73 logs), especially the subunit vaccines without TPPPS, which had significantly lower levels of protection than the *B. melitensis* M5 vaccine (*P* < 0.05). These data demonstrate that the expressed Omp10-Omp28-L7/L12 proteins effectively protected mice against *Brucella* infection, and using TPPPS as an adjuvant can significantly enhance the protection of Omp10-Omp28-L7/L12 subunit vaccines against *Brucella* infection.

**Table 3:** Protection against challenge with *B. melitensis* 16M after immunization with various vaccines.

**Vaccine**	**Log_**10**_ CFU/spleen**	**Log**
	**(mean ± SD)**	**protection**
*B. melitensis* M5	2.12 ± 0.17^c^	2.73
Omp10-Omp28-L7/L12 (*P. pastoris*)	3.36 ± 0.32^b^	1.49
Omp10-Omp28-L7/L12 (*P. pastoris*) + TPPPS	2.44 ± 0.54^c^	2.41
Omp10-Omp28-L7/L12 (*E. coli*)	3.87 ± 0.55^b^	0.98
PBS	4.85 ± 0.45^a^	0.00
Omp10	4.45 ± 0.83^b^	0.40
Omp28	4.28 ± 0.89^b^	0.57
L7/L12	4.16 ± 0.47^b^	0.69
Omp10-Omp28-L7/L12 (*E. coli*) + TPPPS	2.97 ± 0.68^c^	1.88

*Different superscript lowercase letters indicate significant differences among different groups of mice (P < 0.05 estimated using Duncan's multiple-range tests)*.

## Discussion

*Brucella* still poses a significant threat to domestic animals and humans in many countries. At present, the use of live *Brucella* vaccines is limited, so improving the immunization strategy of *Brucella* subunit vaccines is critical to prevent and control the disease. In this study, we fused three protective antigen genes of Omp10, Omp28, and L7/L12 and expressed this fused protein in both eukaryotic and prokaryotic expression systems. The results indicate that the recombinant Omp10-Omp28-L7/L12 subunit antigens induce positive immune responses and protective effects against *Brucella*, and the protective effects and immunogenicity of the recombinant Omp10-Omp28-L7/L12 protein expressed in the eukaryotic system have advantages compared to those of the prokaryotic Omp10-Omp28-L7/L12 recombinant protein.

Subunit vaccines usually contain one or more specific antigenic epitopes, thus, they have affirmative advantages compared to traditional live and inactivated vaccines, such as targeted immunity, improved safety, fast and easy production, low cost, etc. However, in terms of clinical protection, subunit vaccines rarely have an advantage over conventional vaccines because of their single antigen component. Therefore, the development of multivalent subunit vaccines may be an effective way to develop new generation vaccines. The recombinant expression of the fusion gene is a feasible method to increase the number of epitopes, and this can effectively avoid the repetitive work of single epitope expression and recombination. Previous studies have shown that vaccines composed of a single bacterial protein usually cannot prevent *Brucella* infection (49). Notably, some studies have also demonstrated that for the brucellosis vaccine, the use of compound antigens may provide better immune protection. Simborio et al. showed that the combined rOmp (rOmp10, 19, and 28) antigens have good reactivity for diagnosing disease and provide protection against *B. abortus* infection (11); Golshani et al. found that rL7/L12-TOmp31 can induce a strong IgG response compared with a single protein (25); Ghasemi et al. demonstrated that a recombinant antigen containing rOmp31 + rTF provides a high level of protection against *B. melitensis*, which makes it a good candidate for the development of a multivalent subunit vaccine (50). In view of these results, we designed a fusion protein expression system containing multiple protective antigens to prepare the *Brucella* subunit vaccine. Our results indicate that the recombinant Omp10-Omp28-L7/L12 protein can induce protective immune responses and protection against *Brucella*.

Relative to other proteins, the molecular weights of the outer membrane proteins Omp10 and Omp28 and the ribosomal protein L7/L12 of *Brucella* are small, which makes them convenient for tandem expression. Thus, in this study, we used these three antigenic proteins to create fusion proteins. The results of our study suggest that the antibody levels, peripheral blood lymphocyte proliferation, number of CD4^+^ and CD8^+^ T lymphocytes, and cytokine secretion produced by the Omp10-Omp28-L7/L12 recombinant protein vaccinated groups were significantly higher than those of the negative control group, indicating that recombinant protein vaccines can produce specific antibodies in mice and exert good immunogenicity. The detection of lymphocytes and the results of flow cytometry showed that the frequency of CD4^+^ and CD8^+^ T cells increased significantly. This result demonstrated the activation of antigen-specific T cells *in vivo*. Although the importance of these two T cell populations has been disputed, both CD4^+^ and CD8^+^ T cells may be important in immunity against *Brucella* infection. BALB/c mice were infected with *Brucella* and immunized with CD4^+^ and CD8^+^ T cells for 6 weeks, the CFU of *Brucella* in the spleen of the mice was found to be lower than that of untreated mice (51). This showed that both T cells play a role in the immunization of *Brucella*. However, recent experiments have shown that the ability of MHC I-deficient mice (without CD8^+^ T cells function) to control infection after infection with S19 was much lower than that of wild MHC II-deficient mice (without CD4^+^ T cells function). The ability to control infection was similar to that of normal mice (52), indicating that CD8^+^T cell plays a major role; and further experiments indicated that CD8^+^ T cells play a limited role in the early stages of infection. In addition, experiments with MHC I-deficient mice confirmed that CD8^+^ T cells play a major antibacterial role after the peak of infection. However, since the number of CD4^+^ T cells accounts for the vast majority of T cells, and they can also secrete IFN-γ, the ability of CD4^+^ T cells to control *Brucella* cannot be ignored. About the critical role of Th1 and Th2 immune responses in the control of *Brucella* infection, the levels of Th1 cytokine (IL-2 and IFN-γ) and Th2 cytokine (IL-4) from all mice groups were evaluated and enhanced levels of IL-2, IL-4 and IFN-γ (P < 0.05) were observed in comparison to the PBS group. The result of the challenge experiment indicates that immunization of mice with Omp10-Omp28-L7/L12 subunit vaccines results in a significant reduction in the *B. melitensis* burden in the spleens of mice and has good protective effects. Taken together, the recombinant antigen of Omp10-Omp28-L7/L12 elicited a good immune response and could serve as a good candidate for the development of multivalent subunit vaccines that provide high levels of protection against *Brucella*. However, we also found that the protective effect of the recombinant Omp10-Omp28-L7/L12 protein was not as effective as the live vaccine. Other previously reported recombinant proteins also showed similar results (53, 54), which were probably due to the lack of cellular immune response compared to the live vaccine. Considering the advantages and disadvantages of both vaccines, a differentiated *Brucella* immunotherapy is necessary for specific situations in different epidemic areas.

It has been revealed that TPPPS can enhance the stimulation ability of the vaccines and induce better cellular and humoral immunity (39, 40). TPPPS was used as an adjuvant in our research, showed an obvious enhancement in immune response and protection to the recombinant Omp10-Omp28-L7/L12. Although the live vaccine group showed better protection, the difference between the TPPPS adjuvant group and the live vaccine group was not significant. This means that the use of TPPPS as an adjuvant narrows the gap between the recombinant Omp10-Omp28-L7/L12 subunit vaccine and the live vaccine. Therefore, TPPPS can be used as a potential adjuvant for *Brucella* subunit vaccines.

Herein, we used *P. pastoris* eukaryotic and *E. coli* prokaryotic expression systems to express the recombinant Omp10-Omp28-L7/L12 protein. The advantages of the *E. coli* expression system are easy growth and simple gene manipulation, therefore the system is considered to be the most economical, convenient and effective expression system (55). However, a broad understanding holds that *E. coli* lacks proper post-translational processing mechanism leads to vast differences between the expressed bacterial epitopes and its natural structure (56). The *P. pastoris* eukaryotic expression system has some advantages, such as easy culture, high yield, accurate post-translational modification, and low interference of natural proteins, etc. Additionally, this secretory expression system also facilitates subsequent protein purification (57). As expected, the recombinant Omp10-Omp28-L7/L12 was secreted into the medium at a high yield, and the content of natural proteins in *P. pastoris* were very low, which greatly improved the purification efficiency of recombinant fusion protein. Moreover, both the quantity and the immunogenicity of the Omp10-Omp28-L7/L12 recombinant protein expressed in *P. pastoris* were slightly higher than those of the recombinant protein in *E. coli*. From the protection experiment, the recombinant Omp10-Omp28-L7/L12 protein expressed in *P. pastoris* induced a higher level of protection than the recombinant Omp10-Omp28-L7/L12 protein expressed in *E. coli*. And the fusion proteins induced a higher level of protection than the single proteins. Thus, given the protein expression quantity, protein purification, and immune effects, the *P. pastoris* expression system is more suitable for expressing the recombinant fusion protein than the *E. coli* expression system in our study.

## Conclusion

*P. pastoris* eukaryotic and *E. coli* prokaryotic expression systems were used to prepare *Brucella* subunit vaccines in this study. We have demonstrated that recombinant antigen containing Omp10-Omp28-L7/L12 is a good candidate for the development of multivalent subunit vaccines, which provide definite protection against *Brucella*. We also found that the quick performance and immune effects of the recombinant *Brucella* subunit expressed in *P. pastoris* have certain advantages compared with the traditional *E. coli* expression system. Moreover, TPPPS performed as a good adjuvant in the subunit vaccine. Together, the results of this study reveal the potential of the fused multivalent subunit vaccine for the prevention of brucellosis.

## Data Availability Statement

The datasets presented in this study can be found in online repositories. The names of the repository/repositories and accession number(s) can be found in the article/Supplementary Material.

## Ethics Statement

The animal operation procedures were approved by the Animal Care and Use Committee of Shandong Agricultural University (permit number: 20010510) and implemented according to the Guidelines for Experimental Animals of the Ministry of Science and Technology (Beijing, China).

## Author Contributions

RZ, KW, LH, and LZ designed the research. LZ, QW, YW, and YX performed the research. LZ, LH, KW, DP, HH, and RZ analyzed the data. LZ, LH, KW, and RZ wrote the manuscript. All authors contributed to the article and approved the submitted version.

## Conflict of Interest

HH was employed by company Shandong New Hope Liuhe Co., Ltd. HH is one of the inventors of a pending patent (CN-109486846-A) relevant to this work. The remaining authors declare that the research was conducted in the absence of any commercial or financial relationships that could be construed as a potential conflict of interest.

## Supplementary Material

Figure S1 Purification of the fused Omp10 protein (14.6 kDa), Omp28 protein (28 kDa), and L7/L12 protein (14 kDa). M, Page ruler pre-stained protein ladder; lane 1, purified Omp10 protein (*E. coli*); lane 2, purified Omp28 protein (*E. coli*); lane 3, purified L7/L12 protein (*E. coli*).

Table S1 Primers list of genes (*E. coli* prokaryotic expression).

Table S2 Primers list of genes (*P. pastoris* eukaryotic expression).
