# Supplementary figures and images for: Comparison of Immune Effects Between Brucella Recombinant Omp10-Omp28-L7/L12 Proteins Expressed in Eukaryotic and Prokaryotic Systems
Source: Front Vet Sci. 2020 Sep 18;7:576. doi: 10.3389/fvets.2020.00576 (PMC7531237; doi:10.3389/fvets.2020.00576)

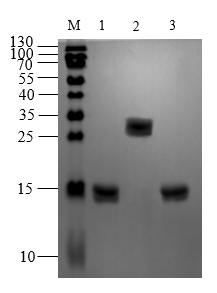

Supplement: Figure S1 — Purification of the fused Omp10 protein (14.6 kDa), Omp28 protein (28 kDa), and L7/L12 protein (14 kDa). M, Page ruler pre-stained protein ladder; lane 1, purified Omp10 protein (E. coli); lane 2, purified Omp28 protein (E. coli); lane 3, purified L7/L12 protein (E. coli). [file Image_1.JPEG]
