# Supplementary material for: Comparison of Immune Effects Between Brucella Recombinant Omp10-Omp28-L7/L12 Proteins Expressed in Eukaryotic and Prokaryotic Systems
Source: Front Vet Sci. 2020 Sep 18;7:576. doi: 10.3389/fvets.2020.00576 (PMC7531237; doi:10.3389/fvets.2020.00576)
Supplement: Table S1 — Primers list of genes (E. coli prokaryotic expression). [file Table_1.DOC]

**Tables**

**Table S1|**

Primers list of genes (*E. coli* prokaryotic [expression](javascript:;)).

| genes | Primers list |
| --- | --- |
| Omp10F  Omp10R-linker | 5'-CGC*GGATCC*ATGAAACGCTTCCGCATCGTT-3' ( *BamH I* site in italics ) |
|  | 5'-GCTTCCTCCTCCTCCGCTTCCTCCTCCTCCGCTTCCTCCTCCTCCGCCGGCGTTGCGGCGGGTG-3' |
| Omp28F-linker  Omp28R-linker | 5'-GGAGGAGGAGGAAGCGGAGGAGGAGGAAGCGGAGGAGGAGGAAGCAACACTCGTGCTAGCAATTTT-3' |
|  | 5'-GCTTCCTCCTCCTCCGCTTCCTCCTCCTCCGCTTCCTCCTCCTCCCTTGATTTCAAAAACGACATT-3' |
| L7/L12F-linker  L7/L12R | 5'-GGAGGAGGAGGAAGCGGAGGAGGAGGAAGCGGAGGAGGAGGAAGCGCTGATCTCGCAAAGATCGTTG-3' |
|  | 5'-CCG*CTCGAG*TTAATGATGATGATGATGATGCTTGAGTTCAACCTTGGCGCCAG-3' ( *Xhol I* site in italics ) |

The long underscores are the gene encoding linker; the short underscores are the gene encoding His tag

**Table S2|**

Primers list of genes (*P. pastoris* eukaryotic [expression](javascript:;)).

| genes | Primers list |
| --- | --- |
| F | 5'-CGC*CTCGAG*ATGAAACGCTTCCGCATC-3' ( *Xhol I* site in italics ) |
| R | 5’-TT*GCGGCCGC*TCAATGATGATGATGATGATGCTTGAGTTCAACCTTGGCGCCAG-3' (*Not I* site in italics ) |
